# Supplementary figures and images for: Landscape conservation and orchard management influence carob tree yield through changes in pollinator communities
Source: PLoS One. 2025 Feb 14;20(2):e0307357. doi: 10.1371/journal.pone.0307357 (PMC11828422; doi:10.1371/journal.pone.0307357)

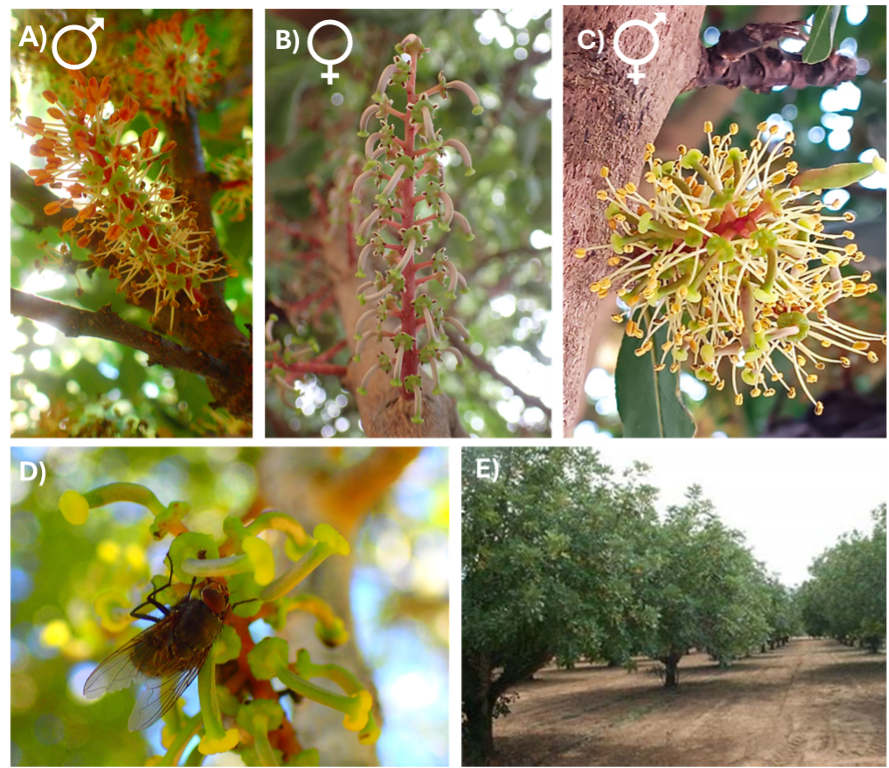

Supplement: S1 Fig — (PNG) [file pone.0307357.s001.png]

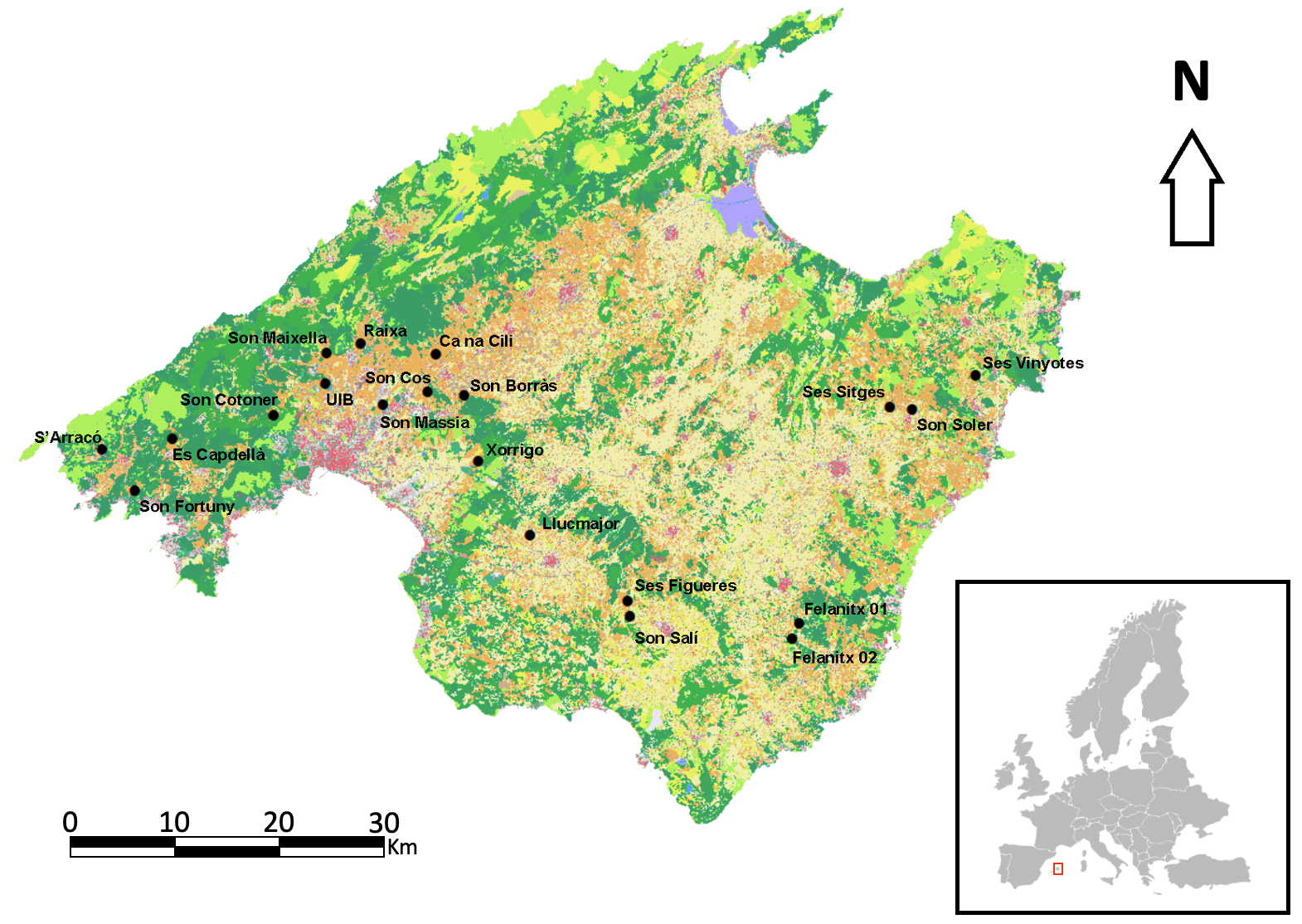

Supplement: S2 Fig — (PNG) [file pone.0307357.s002.png]
