# Supplementary material for: Landscape conservation and orchard management influence carob tree yield through changes in pollinator communities
Source: PLoS One. 2025 Feb 14;20(2):e0307357. doi: 10.1371/journal.pone.0307357 (PMC11828422; doi:10.1371/journal.pone.0307357)
Supplement: Attachment — Submitted filename: PONE-D-24-27278-Peer review.docx [file pone.0307357.s010.docx]

This current version of this study demonstrated that the % natural habitat, overall pollinator abundance and male-to-female ratio significantly affected the seed production of carob trees. Basically, the experimental method is sound, the result is interesting and the discussion is reasonable. However, there are still some issues that need to be revised and adjusted the prior to publication.

1. In the abstract, please clarify the current problems that need to be solved in the production of carob, that is, the purpose of the study.
2. There is a lack of review or comparison of similar studies in the preface, such as: the impact of different landscape types or orchard management on yield? The research value and significance of long-horned beans are not clear.
3. L98～L120: The introduction of research crops is suggested to be included in the preface to comprehensively review the research value and significance of carob。
4. Supplement orchard information (variety, planting area, tree age, flowering, fruiting, climate, elevation, etc.) in material method。
5. L19～L20，L124: Orchard local management (farming system: conventional vs. ecological)’ and ‘Fig. 1’ mention ‘conventional vs. ecological,’ but the specific concepts or definitions related to this are found in lines 153–156. It is suggested to unify and organize the research orchard’s location information, management information, and so on, to enhance the readability of the paper.
6. L129～L130:“it cannot be totally discarded that they include different cultivars，as this information was unknown by the farmers.”. Although different orchards have different varieties, it should be explained here whether the different varieties affect the assessment and comparison of yields between different orchards.
7. L159～L161:“The male-to-female ratio in carob orchards of Mallorca is generally low, as farmers obtain female carob trees by grafting female branches in male rootstocks to increase the number of producer trees”. It is recommended to include this content in the introduction or discussion section for elaboration
8. It is recommended that the author include a diagram illustrating pollinator visits to flowers. This addition will enhance the content of the paper and provide readers with a clearer understanding of carob as an economic crop.
9. L162～L163: “except for one study orchard where most of the individuals (95.7%) were hermaphrodite”. When evaluating the impact of the male-female ratio on yield, should the data from this orchard be excluded? The results will be more accurate and will be more instructive for future carob planting.
10. L195-197：I have a concern: is it sufficient for each tree to randomly select a branch to evaluate yield metrics?
11. L291～L292: According to the result description part “Fruit production per female tree varied between 8.8 and 45.2 fruits in one thousand flowers”, is it more appropriate to change “Fruit production per female tree” to “Number of fruits per female tree”? Seed yield is the same as above. L295 “seed weight” is changed to “seed production”. Like lines L446~L447 (the number of carob seeds, the weight of the seeds), this is easier to understand.
12. Judging from the results in Table 1 A, there is a positive correlation between overall pollinator abundance and farm system, but the relationship is not significant (P=0.097), rather than “the effect was only marginally significant” as the author said.
13. L433～L435:“On the contrary, pollinator communities associated with more disturbed landscapes (high abundance of honeybees) negatively influenced seed weight.”. Does higher bee abundance have a negative effect on seed weight? This conclusion seems inconsistent with common sense? Although wild bees are more efficient pollinators, bees are also efficient pollinators of flowering plants.
