# Supplementary material for: Landscape conservation and orchard management influence carob tree yield through changes in pollinator communities
Source: PLoS One. 2025 Feb 14;20(2):e0307357. doi: 10.1371/journal.pone.0307357 (PMC11828422; doi:10.1371/journal.pone.0307357)
Supplement: Attachment — Submitted filename: PONE-D-24-27278-Peer_review_declet_1.docx [file pone.0307357.s011.docx]

I appreciate the authors’ timely response to my previous comments and their diligent efforts in revising the manuscript. The revised version addresses the concerns raised, demonstrating a strong grasp of the subject matter. The study effectively investigates the relationships between natural habitat, pollinator abundance, and sex ratios in carob tree seed production, which is of significant ecological and agricultural importance.

Overall, I find this revised article to be well-structured, informative, and valuable to the field of agricultural ecology. I recommend its acceptance for publication, as it contributes significantly to our understanding of factors influencing carob seed production and offers insights that could inform future agricultural practices.
