# Supplementary material for: Landscape conservation and orchard management influence carob tree yield through changes in pollinator communities
Source: PLoS One. 2025 Feb 14;20(2):e0307357. doi: 10.1371/journal.pone.0307357 (PMC11828422; doi:10.1371/journal.pone.0307357)
Supplement: Attachment — Submitted filename: ResponseLetter_RIII_AL.docx [file pone.0307357.s012.docx]

**Authors' Responses to Reviewers’ comments**

**Reviewer #1**

I appreciate the authors’ timely response to my previous comments and their diligent efforts in revising the manuscript. The revised version addresses the concerns raised, demonstrating a strong grasp of the subject matter. The study effectively investigates the relationships between natural habitat, pollinator abundance, and sex ratios in carob tree seed production, which is of significant ecological and agricultural importance.

Overall, I find this revised article to be well-structured, informative, and valuable to the field of agricultural ecology. I recommend its acceptance for publication, as it contributes significantly to our understanding of factors influencing carob seed production and offers insights that could inform future agricultural practices.

**REPLY: Thank you for your positive assessment of the manuscript.**

**Reviewer #2**

I appreciate the revision done by the authors, and all my comments were taken into account or explained/counter discussed. I have minor comments to give upon the new read of the document, but I think the manuscript is ready for publication. Congrats on the work.

**REPLY: Thank you very much for your revision. Below you will find our replies to your comments.**

Line 72 – 79: Here, I miss the indication that equilibrated sex ratios are also needed so that suitable levels of pollen (from the males) are available for fertilization of the female flowers (maybe it could be in the line before, 71-72). I know it is obvious for researchers with knowledge in this type of crop, but it would be nice the be stated.

**REPLY: We added this explanation to the sentence as suggested (lines 72-75).**

Line 100-102: Can you revise this phrase, please? It becomes confusing the influence of the effects on pollinators. Maybe it could be improved.

**REPLY: We have rewritten this sentence according to your suggestion (lines 101-104).**

Line 103-104: “Understanding how the landscape context and local management of orchards affect carob tree yields …”. This is a repetition of the previous phrase, maybe rephrase to avoid repetition.

**REPLY: We slightly modified the sentence to avoid repetition with the previous one (lines 104-106).**

Line 133: I suggest 1.50 km to maintain the number of decimals.

**REPLY: Done (line 133)**

Line 214: In/For both study years.

**REPLY: Added (line 214)**

Line 312: See Figures.

**REPLY: This is not a typo, “Ses Figueres” is the name of one of the study sites, as is “Son Cotoner” that it is named in the same sentence. We have added “” to clarify that these are names of study sites (lines 311-312)**

Line 315: I suggest naming honeybees, once it was the name appointed in the methods section on line 205.

**REPLY: Changed (line 315)**

Line 328-329: Similar here to the other flies.

**REPLY: We added “other flies” together with the description “(muscoid and small acalyptrate flies)” (line 328-329)**

**Reviewer #3**

I want to thank the authors for their efforts in revising their paper, they have done an excellent job incorporating the comments of all the reviewers to produce a significantly improved manuscript.

I am satisfied with the additional information and explanation added about honey bee hives on Mallorca, and thank the authors for providing the further information! I also thank them for conducting the additional spatial autocorrelation analysis, which has strengthened the validity of their results. I also appreciate the inclusion of photos of the inflorescences as recommended by another reviewer, it’s nice have a visual of the system (as someone who’s never seen a carob tree!)

I only have a very few small grammatical edits I noticed (see below), otherwise I recommend this paper for publication upon making these last small changes.

**REPLY: Thank you for your valuable first revision, which helped us improve the manuscript, and for your help with the grammatical edits now.**

Line 67: It’s very unusual to start a paragraph with “besides,” I would recommend taking it out and adding “also” later in the sentence (and making effect plural): “The effects of pollinator loss on crops might also depend[…]”

**REPLY: Done (line 67)**

Line 83: Needs slight rewording to make sense, my suggestion would be: “[…] clinical applications, with the seeds being the most profitable […]”

**REPLY: Done (line 84)**

Line 100: This should either say “might lead to spatial isolation” or “might drive spatial isolation”

**REPLY: Done (line 101)**

Line 147: small typo, should say “independent of whether”

**REPLY: Changed (line 147)**

Line 170: add “and” after the comma (“and the male-to-female ratio”)

**REPLY: Done (line 170)**

Line 227: Should be “weighing” (not weighting)

**REPLY: Done (line 227)**

Line 250: Should be past tense “ran” (instead of run)

**REPLY: Done (line 250)**

Line 533: Using “On the contrary” is usually used to negate a statement in the sentence immediately before, which was not your intention here (I think you mean to contrast the statement two sentences back) - I would recommend changing to “However,” or “Contrary to our prediction, […]” instead.

**REPLY: Done (line 533)**
